# Generation of a Well-Characterized Homozygous Chromodomain-Helicase-DNA-Binding Protein 4^G1003D^ Mutant hESC Line Using CRISPR/eCas9 (ULIEGEe001-A-1)

**DOI:** 10.3390/ijms241310543

**Published:** 2023-06-23

**Authors:** Ilyas Chohra, Subhajit Giri, Brigitte Malgrange

**Affiliations:** Developmental Neurobiology Unit, GIGA-Stem Cells, Av Hippocrate, 15 B-4000 Liege, Belgium; ilyas.chohra@uliege.be

**Keywords:** cochlea, hair cells, epigenetic, differentiation, development, CRISPR, CHD4

## Abstract

The chromatin remodeler Chromodomain-helicase-DNA-binding protein 4 (CHD4) is crucial for the development of multiple organ systems. Functional mutations of CHD4 have recently been described in a developmental disorder, namely Siffrim-Hitz-Weiss syndrome (SIHIWES). Herein, we have generated a homozygous CHD4G1003D hESC line (WAe025-A-1) using CRISPR/eCas9-based gene editing in the WA-25 hESC line. The edited hESC line maintains normal karyotype, pluripotency, and ability to differentiate into three germ layers. This cell line will be a valuable resource for studying the functional role of CHD4 during the development and disease modeling of SIHIWES in vitro.

## 1. Introduction

The ATP-dependent chromatin remodeler CHD4 is a core component of the nucleosome remodeling and histone deacetylation (NuRD) complex and an essential epigenetic factor to regulate spatiotemporal gene expression during development [1,2]. The ATP-dependent chromatin remodeler proteins are implicated in many developmental disorders, including hearing loss [2,3]. Normal CHD4 activity was found to be essential for maintaining stem cell pluripotency [4], differentiation capacity towards neuronal lineage [5], T cell development and hematopoietic stem cell [6,7], cardiac development [8,9], renal progenitor cell self-renewal, and differentiation [10]. It has also been shown that CHD4 can regulate gene expression for maintaining a distinct cellular state and pledge for accurate cell fate decisions upon developmental/external cues [11]. CHD4 was shown to be very important during mammalian embryogenesis as it helps in the formation of functional trophectoderm and lineage commitment of unspecified cells at the preimplantation stage by regulating the frequency of lineage-specification gene expression [12]. Recently, de novo mutations in CHD4 have been implicated in a distinct developmental disorder, ‘Sifrim-Hitz-Weiss’ (SIHIWES) syndrome [13]. De novo mutations in CHD4 were also found in syndromic and non syndromic congenital heart defect patients and moyamoya angiopathy [14,15]. To study the role of wild-type and mutant CHD4 protein in development and disease, we have generated a mutant CHD4 (c.3008G>A; p. G1003D) hESC line and performed functional characterization. This CHD4^G1003D/G1003D^ hESC model would provide ample opportunity to differentiate towards targeted cell types in 2D and 3D organoids and study the functional deficit of the specific CHD4 mutant version in cardiac, retinal, neuronal, skeletal, gonadal, and auditory system development.

**Figure 1:**
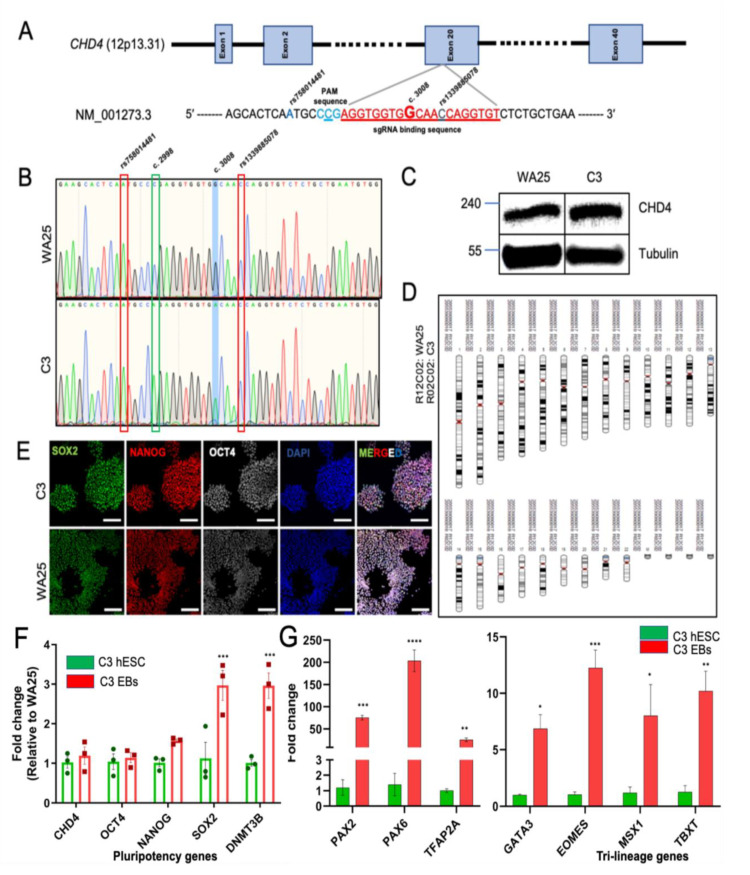
Generation and characterization of WA25-CHD4G1003D/G1003D hESC line. (**A**) Illustration of CRISPR experiment design to single base editing at c.3008G>A. The gRNA target sequence is indicated in red color followed by the PAM sequence in blue; the c.3008G is in bold, and the silent mutation to alter the PAM site is underlined; the two SNP markers flanking the desired insertion site are indicated by blue color. (**B**) Sanger sequence chromatogram of wild type WA25 hESC and edited C3 hESC line. Highlighted in blue is the position of the desired base change (c.3008G>A). (**C**) Western blot images of wild type CHD4 and mutant CHD4 (p. G1003D) proteins (upper panel) extracted from WA25 hESC and C3 line. The lower panel is for loading control α- tubulin. (**D**) Karyotype of WA25 (R12C02) and C3 (R02C02) hESC line as visualized through KaryoStudio v1.4 software. (**E**) Immunofluorescence imaging of pluripotency markers OCT4, NANOG and SOX2 for both WA25 and C3 hESC colonies. Images were captured in Zeiss LSM 980 confocal microscope with 20X objective, Z-stacked with 1.3 µm optical slices. Images were processed in ImageJ software (scale bar = 100 µm). (**F**) qPCR-based quantification of pluripotency marker gene expression in WA25 and C3 hESC lines, statistical analysis was performed as 2-way ANOVA with Sidak’s test, *—*p* < 0.05, **—*p* < 0.01, ***—*p* < 0.001, ****—*p* < 0.0001, ns—*p* > 0.05. (**G**) qPCR based quantitative analysis of three germ layer marker gene expression (Ectoderm: PAX2, PAX6, TFAP2A; Endoderm: GATA3, EOMES, and Mesoderm: MSX1, TBXT) in C3 hESC derived embryoid bodies compared to the C3 pluripotent stages.

Here, we have targeted exon 20 of CHD4, specifically at c. 3008G through CRISPR/eSpCas9 system and allowed the precise insertion of c. 3008A by favoring the homologous recombination (HR) mediated DNA repair (Figure 1A, the single guide RNA (sgRNA) target sequence is indicated in red, followed by the protospacer adjacent motif (PAM) sequence in blue; the c.3008G is in bold, and the silent mutation to alter the PAM site is underlined; the two single nucleotide polymorphism (SNP) markers flanking the desired insertion site are indicated by blue color). This in vitro hESC model can help study the role of CHD4 mutation during the development of the ‘Sifrim-Hitz-Weiss’ (SIHIWES) syndrome. The targeted differentiated cell types and organoids for cardiac, renal, ocular, and neuronal systems will provide a unique opportunity to study the functional role of CHD4 in the development and related disorders.

## 2. Results

### 2.1. Efficient Gene-Editing Generates Multiple Heterozygous and Homozygous Single-Cell Derived Mutant Clones

The WA25 hESCs were nucleofected with sgRNA oligo cloned pLentiCRISPR-E (Addgene #78852) and DNA repair template single-stranded oligonucleotide (ssODN), and subjected to puromycin selection for positively transfected cells. The puromycin-resistant single-cell derived colonies were manually picked up and expanded in two separate batches. We have picked around 160 colonies for further analysis. These colonies are morphologically pluripotent in nature, devoid of any spontaneous differentiation, and well-separated from surrounding colonies. this is confirmed further (Table 1). Genomic DNA samples were isolated from each colony from one batch and genotyped through PCR, restriction fragment length polymorphism (RFLP) assay, and Sanger sequencing (primer sequences are listed in Table 2). We have identified 14 heterozygous *CHD4* c.3008G>A clones and one clone with homozygous insertion of c.3008A, as confirmed by the Sanger sequencing chromatogram (Figure 1B). That made our CRISPR experiment 9.37% efficient for homology-directed repair (HDR). As we envision to delineate the functional role of CHD4^G1003D^ homozygous mutant in development and disease, we only further characterize the homozygous mutant clone and banked the heterozygous clones. The *CHD4* c.3008G>A (homozygous) cell line [WA25-*CHD4*^c.3008A/A^ (C3) and mentioned as C3 henceforth] was expanded for further characterization and validation (Table 1).

### 2.2. C3 Line Maintains Pluripotency and Normal Karyotype

To find out if c.3008G>A mutation has any effect on CHD4 RNA and protein expression, we assessed the CHD4 p. G1003D homozygous mutant protein expression level through immunoblotting along with the wild-type CHD4 protein and found no significant change in protein expression (Figure 1C). At the RNA level, mutant CHD4 has also comparable expression like wild-type CHD4 mRNA as shown through qPCR analysis (Figure 1F). The chromosomal integrity of C3 cells was confirmed by digital karyotyping through whole-genome SNP genotyping and copy number variation analysis, which found a normal female karyotype without any chromosomal aberration (Figure 1D, Supplementary Figure S1). In Figure 1D, the X-chromosome images are not evident as the KaryoMap was obtained from KaryoStudio v1.4 (Illumina). There is a ‘software bug’ in KaryoStudio v1.4, which prevents the software drawing the X/Y chromosome in the KaryoMap. To circumvent this issue, we have provided Supplementary Figure S1, where the B-allele frequency and LogR ratio for X-chromosome is depicted for both the WA25 and C3 hESC lines. This dataset also showed the absence of a Y chromosome, proving the C3 is a WA25 cell of origin (Supplementary Figure S1). Positive immunostainings (>95%) against standard pluripotency markers SOX2, NANOG, and OCT4, as well as a tightly packed morphology of C3 cells, confirmed the pluripotent characteristics comparable with wild type WA25 hESCs (Figure 1E, Supplementary Figure S2). RNA Expression levels of pluripotent genes (*OCT4*, *NANOG*, *SOX2*, and *DNMT3B*) have been analyzed by qRT-PCR (Figure 1F). The expression of *OCT4* and *NANOG* was found to be comparable to that of WA25. In contrast, *SOX2* and *DNMT3B* expressions were found to be significantly higher in C3 cells. This can be explained by the fact that CHD4 deficiency has previously been found to affect the pluripotency gene expression [4]. SOX2 and DNMT3B genes are direct targets of CHD4, and the p. G1003D mutation could impair the repressive activity of CHD4 on *SOX2* and *DNMT3B* expression.

### 2.3. C3 Line Is Able to Differentiate into Three-Germ Layers

The ability of the C3 line to differentiate into three germ layers was assessed by in vitro embryoid body (EB) formation followed by three germ layers markers expression analysis by qRT-PCR and compared with the expression levels of those markers in the C3 pluripotent stage (Figure 1G). We have found that the ectodermal markers (*PAX2*, *PAX6,* and *TFAP2A*), endodermal markers (*GATA3*, *EOMES*) and mesodermal markers (*MSX1*, *TBXT*) significantly upregulated in expression compared to that of C3 pluripotent cell population. The significant fold-change in the expression (2-way ANOVA with Sidak’s test, *—*p* < 0.05, **—*p* < 0.01, ***—*p* < 0.005, ****—*p* < 0.001) of the marker genes confirmed that the C3 hESC line is capable of differentiating into three germ layers confirming its pluripotency characteristic again.

### 2.4. C3 Line Qualified through Several Additional Quality Control Experiments

We have first checked for any undesired off-target editing that happened in C3 line. The ‘CCTop’ algorithm predicted the top five exonic off-targets were screened for potential off-target editing through targeted PCR followed by Sanger sequencing and they were found to be unaffected in all five genomic sites (Supplementary Figure S4). Luminescence-based mycoplasma testing confirmed that the C3 cell line is free from any mycoplasma infection (Supplementary Figure S3). Cell type identity of C3 and WA-25 hESCs were validated by STR analysis of 33 loci (archived with journal).

## 3. Discussion

The CHD4 protein belongs to the SNF2/RAD54 helicase family and is one of the core components of NuRD complex [1,16,17]. CHD4 also belongs to CHD subfamily II and characteristically contains PHD fingers and chromodomains as well as an SNF2-like ATPase/helicase core [18]. The PHD and chromodomains were found to bind nucleosome and histone H3 tail to regulate the ATPase and chromatin remodeling activity [19,20]. CHD4 has a ubiquitous expression and primarily acts as a transcriptional repressor [21,22,23]. CHD4 protein activity is involved in certain key developmental pathways like spermatogenesis and gonadal development [24,25,26], neuronal and brain development [27,28], cardiac development and maintenance [8,9,29], and vascular development [30,31,32]. So, it is conceivable that any pathogenic loss-of-function or gain-of-function mutations and functional impairment of CHD4 activity would severely impact the spatiotemporal transcriptional network and normal developmental pathways.

The G1003D mutation was found in an adolescent-onset male patient affected with ‘Siffrim-Hitz-Weiss’ syndrome with multiple organ systems affected [13]. Phenotypically, the patient has a developmental delay with delayed speech and learning difficulties in school, hypotonia, and mild intellectual disability. The brain MRI identified an enlarged lateral and third ventricle. The patient developed glaucoma and sensorineural hearing loss. His cardiac system is also affected by atrial septal defect, patent ductus arteriosus, and ventricular septal defect. The patient has micropenis with delayed sexual development with decreased gonadotropin level. As other phenotypic features, the patient has a short stature without growth hormone deficiency, hypertelorism, a squared face, widow’s peak, skeletal osteopenia, short palpebral fissures with microcornea, small ears with dysmorphic helices, and joint hyperlaxity. A singleton, whole genome sequencing identified only CHD4 c.3008G>A as a candidate pathogenic mutation. It is evident from this study that CHD4 p.G1003D pathogenic mutation affects significantly the normal CHD4 activity during development alongside other pathogenic mutations. In a recent study, Cryo-Electron microscopy-based structural analysis at a 1.3 Å resolution was undertaken to determine the nucleosome-CHD4 complex interaction [33]. Structurally, the G1003 residue is located in ATPase lobe 2 of the CHD4 protein. The G1003D mutation of CHD4 was shown to disrupt the interaction with H3 alpha helix loop [33]. Loss of such interaction or deletion of the loop in closely related protein CHD1 was shown in a loss of chromatin remodeling activity [34], which may stand true for CHD4 also.

With the advent of hESC/hiPSC derived targeted cell type differentiation in 2D and 3D organoid, microfluidic-based organ-on-a-chip/disease-on-a-chip engineering, the understanding of disease mechanism and drug discovery against developmental disorders gained an unprecedented boost [35,36,37]. To complement such efforts, parallel development of appropriate disease models with hESC/hiPSC derived organoids has a pivotal importance [38,39,40]. Here, we have generated a well-characterized clinically relevant CHD4^G1003D^ homozygous mutant hESC line to contribute to the effort of disease modeling of SIHIWES.

## 4. Materials and Methods

### 4.1. Cell Culture

The WA-25 hESC line was cultured on Vitronectin (Gibco™, # A31804) coated plates (Corning) using mTeSR™ Plus media (STEMCELL™ Technologies, # 100-0276) under standard cell culture conditions (37 °C, humidified air with 5% CO_2_). Cell colonies were routinely checked for intact pluripotent stage morphologies and the presence of any random differentiation, and if any, mechanically cleaned immediately. Cells were passaged with 0.05 mM EDTA solution at around 80% confluency in a 3–5 cell micro-colony format.

### 4.2. Precise Gene Editing with CRISPR/eCas9-Based HDR Repair

CRISPR sgRNA oligos were designed using the ‘CCTop’ algorithm (https://crispr.cos.uni-heidelberg.de/ (accessed on 22 May 2023)), targeting the c. 3008 G (NM_001273.3) at exon 20 of the CHD4 gene. Synthesized sgRNA oligos were cloned into pLentiCRISPR-E (Addgene #78852) containing eSpCas9 and a puromycin selection cassette in a lentiviral vector backbone. The sequence-verified successful sgRNA/eSpCas9 clone was purified by EndoFree^®^ Plasmid Maxi Kit (Qiagen #12362). For the HDR template, a 101 base long ssODN template was designed having the desired base change c. 3008 G>A and a ‘codon usage bias’ optimized PAM altering c. 2998 C>A silent mutation. Before nucleofection, WA-25 cells were treated with 200 nM Nocodazole (STEMCELL™ Technologies, # 74072) to synchronize the cells in the G2 phase, as the HDR happens more frequently in G2 cell cycle phase, and RevitaCell™ Supplement (Gibco™, # A264450) for 16 h. The treated WA-25 cells were dissociated with Accutase™ solution (STEMCELL™ Technologies, # 07920), and 1 × 10^6^ live cells were nucleofected with P3 Primary Cell 4D-Nucleofector^®^ X Kit (Lonza # V4XP-3024) and Amaxa^®^ 4D Nucleofector ^®^ (Lonza # AAF-1003X) following optimized CA-137 program and the manufacturer’s protocol. A total of 4 μg of sgRNA/eSpCas9 construct and 1 μM of ssODN were nucleofected. Immediately after nucleofection, cells were plated on a vitronectin-coated 6-well plate with a density of 1.66*10^5^ cells/well using pre-warmed mTeSR plus media supplemented with Revitacell™. Post 24 h of recovery, cells were subjected to puromycin (Sigma #P8833-10MG) selection for positive transfection using 0.5 µg/mL puromycin and Revitacell™-supplemented mTeSR plus media. To favor the HR-based repair, 1 μM Scr7 (DNA Ligase IV inhibitor/NHEJ inhibitor) was used during puromycin selection. Puromycin selection was continued for 48 h with the everyday change of supplemented media, and then the positively selected single-cell derived colonies were expanded for another 10 days with unsupplemented media. Colonies were manually picked and seeded on 96-well plates and allowed to grow until 70% confluency. Each colony was then passaged to duplicate, where one batch was used for genotyping and the other was maintained in culture.

### 4.3. Genotyping through RFLP and Sanger Sequencing

The genomic DNA of each clone was isolated using 50 μL of QuickExtract™ DNA Extraction Solution (Lucigen # QE09050) following the manufacturer’s protocol and PCR was performed with Taq DNA polymerase (Qiagen #201203) using the pre-optimized protocol. PCR amplicons were then subjected to RFLP with Tsp45I restriction enzyme (NEB # R0583L) where the bi-allelic edited product has one cut site to generate 341 and 280 bp long fragments. In contrast, the non-edited product stayed undigested (621 bp). The purified PCR amplicons of positively edited clones were then bi-directionally sequenced using BigDye^®^ Terminator v3.1 sequencing kit (Applied Biosystems^TM^) at the GIGA-Genomics platform (https://www.gigagenomics.uliege.be/cms/c_4164592/en/gigagenomics (accessed on 22 May 2023)). Sequence chromatograms were analyzed by SnapGene Viewer (GSL Biotech) software. Primer and ssODN sequences are provided in Table 2.

### 4.4. Mycoplasma Detection

Mycoplasma testing was performed using MycoAlert™ Mycoplasma Detection Kit (Lonza) at the GIGA-Viral Vectors platform (https://www.gigaviralvectors.uliege.be/cms/c_4215539/en/gigaviral (accessed on 22 May 2023)). A luminescence ratio <1 was considered negative for mycoplasma infection.

### 4.5. Karyotyping and STR Analysis

The chromosomal integrity of edited cells was assessed with digital karyotyping using Illumina Infinium OmniExpress-24 v1.3 Beadchip for whole genome SNP genotyping at the GIGA-Genomics platform. SNP genotyping data were analyzed and visualized by Illumina KaryoStudio Software v1.4. Copy number variation (CNV) was called through B-allele frequency and Smoothed LogR ratio. Karyomap visualization showed no gross chromosomal structural alteration.

The WA-25 and C3 cell lines were authenticated by STR analysis for 33 STR loci. The STR analysis was performed by the Spits group at Reproduction and Genetics Research, Vrije Universiteit Brussels (VUB).

### 4.6. Immunocytochemistry and Western Blot

WA-25 and C3 cells were grown on vitronectin-coated glass coverslips in a 24-well culture plate and fixed with 4% paraformaldehyde (PFA) solution, permeabilized with 0.25% TBS/Triton X-100, blocked by 10% donkey serum (Sigma # D9663-10M) in PBS-T (0.1% Tween 20), and incubated with primary and secondary antibodies (listed in Table 2). Nuclear counterstaining was performed using Hoechst 33342 (CST # 4082S). Images were acquired with Confocal Nikon A1R hybrid resonant microscope and processed and analyzed by ImageJ software.

For Western blots, 250 μL of cell lysis buffer (50 mM Tris-Cl, 150 mM NaCl, 5 mM MgCl2, 1% NP-40, 1 mM DTT, and 1X Protease/Phosphatase inhibitor) was used for each cell line confluent in 35 mm culture dish. Cell lysates were centrifuged at 12,000× *g* for 30 min at 4 °C and protein samples were quantified by Pierce™ BCA assay kit (Thermo Scientific™ #23225). From each sample, 30 μg of protein was separated by NuPAGE™ 4–12%, Bis-Tris gel (Thermo Scientific™ #NP0322BOX), transferred onto a PVDF membrane (Amersham™), and probed with primary and secondary HRP conjugated antibodies (as listed in Table 2). Blots were developed with Pierce™ ECL Western Blotting Substrate (Thermo Scientific™ # 32106). Images were acquired with Amersham™ Imager 600 (GE Healthcare) and analyzed with ImageJ software.

### 4.7. Embryoid Body (EB) Formation and Tri-Lineage Differentiation Assay

The EB generation protocol was adapted from a previously reported one with slight modifications [41]. In brief, cells were dissociated with Accutase™ solution into the single-cell format, and 3 × 10^6^ cells were added to each well of 24-well AggreWell™800 Culture Plates STEMCELL™ Technologies, # 34850) pretreated with Anti-Adherence Rinsing Solution (STEMCELL™ Technologies, #07010). The seeding media used here was mTeSR plus supplemented with 10 μM ROCK-inhibitor (Y-27632) (Selleckchem, # S1049) and 100 μg/mL Normocin™ (InvioGen, # ant-nr-1). After 48 h, the spheroids were transferred to Corning^®^ Costar^®^ Ultra-Low Attachment 6-well plates (Millipore-Sigma, # CLS3471) and cultured in suspension using EB media [Knock-out DMEM (Gibco™ #10829018), 20% KnockOut™ Serum (Gibco™ #10828028), 2 mM GlutaMax (Gibco™ #35050061), 1% PenStrep (Gibco™ #15140122), 0.1 mM NEAA (Gibco™ #11140050), and 0.1 mM β-Mercaptoethanol (Gibco™ #21985023)] for 7 days with orbital shaking. Then, the EBs were transferred to a Matrigel^®^ GFR (Corning^®^ #354230) coated plate and cultured with EB media for another 7 days with media changed every other day. The 3D spheroids were disseminated to 2D three germ layer cells through random differentiation.

### 4.8. RNA Isolation and qPCR Analysis

Total RNA from WA-25, C3 hESCs, and EBs was isolated using TRIzol™ Reagent (Invitrogen™ #15596026) following the manufacturer’s protocol. 1 μg of total RNA from each sample was subjected to cDNA synthesis using RevertAid First Strand cDNA Synthesis Kit (Thermo Scientific™ # K1622) and random hexamer following the manufacturer’s protocol. qPCR was performed using GoTaq^®^ qPCR master mix (Promega # A6002), 1 μL of 1:10 diluted cDNA samples, and pre-validated qPCR primer pairs on a LightCycler^®^ 480 System (Roche). The qPCR data was calculated based on the 2^−∆∆Ct^ method; statistical analysis and graphs were performed using GraphPad Prism v.7 software.

## 5. Conclusions

In a nutshell, we have generated a CHD4 homozygous mutant hESC cell line through CRISPR-based gene editing, which preserved normal karyotype and pluripotency characteristics with the ability to differentiate into three germ layers, which could be a valuable resource to study the pathophysiology of SIHIWES syndrome in vitro.

## Figures and Tables

**Table 1:** Characterization and validation of the new line.

Classification (Optional Italicized)	Test	Result	Data
Morphology	Photography	Normal	Supplementary Figure S3
Pluripotency status evidence for the described cell line	Qualitative analysis (Immunocytochemistry)	Assess staining/expression of pluripotency markers: OCT3/4, NANOG, SOX2	Figure 1E
	Quantitative analysis RT-qPCR Immunocytochemistry counting	1. Positive for OCT3/4, NANOG, SOX2, DNMT3B 2. OCT3/4: 97%; NANOG: 96%; SOX2: 99%.	Figure 1 panel F Figure 1 panel E
Karyotype	Karyotype (higher-resolution, array-based assays (Whole genome SNP genotyping)	46XX, Resolution 50 bps	Figure 1 panel D Supplementary Figure S1
Genotyping for the desired genomic alteration/allelic status of the gene of interest	PCR across the edited site or targeted allele-specific PCR	PCR + Sanger sequencing	Figure 1 panel B
	Evaluation of the—(homo-/hetero-/hemi-) zygous status of introduced genomic alteration(s)	Amplification of a genomic region incorporating at least one adjacent SNP present in the parental line	Figure 1 panel B
	Transgene-specific PCR (when applicable)	N/A	N/A
Verification of the absence of random plasmid integration events	PCR detection specific for plasmid backbones	Negative	N/A
Parental and modified cell line genetic identity evidence	STR analysis	STR analysis of 33 loci, all matched.	Data available upon request
Mutagenesis/genetic modification outcome analysis	Sequencing (genomic DNA PCR)	NM_001273.3 c. 3008 G>A (Homozygous)	Figure 1 panel B
	PCR-based analyses	N/A	N/A
	Western blotting	Comparable expression of mutant CHD4 G1003D protein with wil-type CHD4 protein	Figure 1 panel C
Off-target nuclease activity analysis	PCR across top 5/10 predicted top likely off-target sites	Demonstration of the lack of NHEJ-caused mutagenesis in the top predicted off-target Cas nuclease activity	Supplementary Figure S3
Specific pathogen-free status	Mycoplasma	Mycoplasma testing by luminescence. Negative	Supplementary Figure S3
Multilineage differentiation potential	Embryoid body formation	Expression of three germ layer marker genes Ectoderm: PAX2, PAX6, TFAP2A Endoderm: GATA3, EOMES Mesoderm: MSX1, TBXT	Figure 1 panel G

**Table 2:** Reagent details.

**Antibodies and Stains Used for Immunocytochemistry/Flow-Cytometry**			
	**Antibody**	**Dilution**	**Company Cat # and RRID**
CHD4 (Western Blot)	Mouse anti-CHD4	1:1000	(Abcam Cat# ab70469, RRID:AB_2229454)
α-Tubulin (Western Blot)	Rabbit anti- α-Tubulin (HRP conjugated)	1:5000	(Cell Signaling Technology Cat# 9099, RRID:AB_10695471)
Pluripotency Markers (Immunofluorescence)	Rabbit anti-OCT4	1:300	(Cell Signaling Technology Cat# 2890, RRID:AB_2167725)
Pluripotency Markers (Immunofluorescence)	Mouse anti-SOX2	1:500	(BD Biosciences Cat# 561469, RRID:AB_10694256)
Pluripotency Markers (Immunofluorescence)	Goat anti-NANOG	1:250	(R and D Systems Cat# AF1997, RRID:AB_355097)
Secondary antibodies (Western Blot)	Goat anti-Mouse IgG (H + L) HRP conjugated	1:5000	(Thermo Fisher Scientific Cat# 31430, RRID:AB_228307)
Secondary antibodies (Immunofluorescence)	Donkey anti-Mouse IgG (H + L) Alexa Fluor™ 488	1:500	(Thermo Fisher Scientific Cat# A-21202, RRID:AB_141607)
Secondary antibodies (Immunofluorescence)	Donkey anti-Goat IgG (H + L) Alexa Fluor™ 555	1:500	(Thermo Fisher Scientific Cat# A-21432, RRID:AB_2535853)
Secondary antibodies (Immunofluorescence)	Donkey anti-Rabbit IgG (H + L) Alexa Fluor™ 647	1:500	(Thermo Fisher Scientific Cat# A-31573, RRID:AB_2536183)
Nuclear stain	Hoechst33342	1 µg/mL	Cell Signalling Technology cat # 4082
**Site-Specific Nuclease**			
Nuclease information	eCas9	pLentiCRISPR-E (Addgene #78852; RRID: Addgene_78852) https://www.addgene.org/78852/ (accessed on 12 October 2019).	
Delivery method	Nucleofection (CA137)	P3 Primary Cell 4D-Nucleofector^®^ X Kit (Lonza # V4XP-3024)	
Selection/enrichment strategy	Puromycin/0.5 μg/mL for 48 h post nucleofection		
**Primers and Oligonucleotides Used in This Study**			
	**Target**	**Forward/Reverse Primer (5′-3′)**	
Pluripotency Markers (qPCR)	OCT4	CCCCAGGGCCCCATTTTGGTACC/ACCTCAGTTTGAATGCATGGGAGAGC	
	NANOG	AAAGAATCTTCACCTATGCC/GAAGGAAGAGGAGAGACAGT	
	SOX2	TTCACATGTCCCAGCACTACCAGA/TCACATGTGTGAGAGGGGCAGTGTGC	
	DNMT3B	GAATTACTCACGCCCCAAGGA/ACCGTGAGATGTCCCTCTTGTC	
	REX1	GCCTTATGTGATGGCTATGTGT/ACCCCTTATGACGCATTCTATGT	
Differentiation Markers (qPCR)	PAX2	TGTGGACAGTTTGCGGAAGCA/TGATGTGCTCTGATGCCTGGAA	
	PAX6	CTGAGGAATCAGAGAAGACAGGC/ATGGAGCCAGATGTGAAGGAGG	
	TFAP2A	TAAAGCTGCCAACGTTACCC/GCACACGTACCCAAAGTCC	
	GATA3	CACGGTGCAGAGGTACCC/AGGGTAGGGATCCATGAAGCA	
	EOMES	CAACATAAACGGACTCAATCCCA/ACCACCTCTACGAACACATTGT	
	MSX1	TCCGCAAACACAAGACGA/ACTGCTTCTGGCGGAACTT	
	TBXT	TATGAGCCTCGAATCCACATAGT/CCTCGTTCTGATAAGCAGTCAC	
House-Keeping Genes (qPCR)	GAPDH	GCTCAGACACCATGGGGAAGG/GGAATTTGCCATGGGTGGAATC	
Genotyping (desired allele/transgene presence detection)	N/A	N/A	
Targeted mutation analysis/sequencing	Sequencing data from both alleles	Fwd: GGCCTGAAGAAACCTGATG/ Rev: ACTGTTCCCTAAAGCTCCC	
Potential random integration-detecting PCRs	Plasmid backbone (F/R)	TACAATCTGCTCTGATGCCG/GCTATGTGGCGCGGTATTAT	
gRNA oligonucleotide	sgRNA oligos	Oligo 1: ACACCTGGTTGCCACCACCT Oligo 2: AGGTGGTGGCAACCAGGTGT	
Genomic target sequence(s)	CHD4	NM_001273.3, exon 20	
Bioinformatic gRNA on- and -off-target binding prediction tool used, specific sequence/outputs link(s)	CCTop	https://cctop.cos.uni-heidelberg.de:8043/ (accessed on 22 May 2023).	
Primers for top off-target mutagenesis predicted site sequencing (for all CRISPR/Cas9, ZFN and TALENs)	OT1 (C2orf78)- F/R	TAAAAAGCCCCGAAGCTCCC/GCAGATTGAGGGACAGTAGG	
	OT2 (HCK)- F/R	CTCTAAGCGGGAGGAAAAAC/GGAAGGACAGGAAAACCAAC	
	OT3 (AATK)- F/R	TCCCTCATCTATGCTCTGCC/TGAGCACAAACTCAGGGGAC	
	OT4 (PCK2)- F/R	CACCATCTTCCTGACAATCC/CCCCCCACCTCATAATAACC	
	OT5 (H TMEM231)- F/R	ACCTGTTTAGGGACTTGGTG/TTAAAGAGGGCGGTAGGGAG	
ODNs/plasmids/RNA molecules used as templates for HDR-mediated site-directed mutagenesis.	101 base long ssODN (without any modification)	CAAGTACATCCTCACTCGAAATTTTGAAGCACTCAATGCCAGAGGTGGTGACAACCAGGGTCTCTGCTGAATGTGGTGATGGATCTTAAGAAGTGCTGCA	

## Data Availability

Not applicable.

## Supplementary Materials

The following supporting information can be downloaded at: https://www.mdpi.com/article/10.3390/ijms241310543/s1.
