# Supplementary material for: Generation of a Well-Characterized Homozygous Chromodomain-Helicase-DNA-Binding Protein 4G1003D Mutant hESC Line Using CRISPR/eCas9 (ULIEGEe001-A-1)
Source: Int J Mol Sci. 2023 Jun 23;24(13):10543. doi: 10.3390/ijms241310543 (PMC10342000; doi:10.3390/ijms241310543)
Supplement: Supplementary file 1 [file ijms-24-10543-s001.zip › ijms-2444738-Figure S1.pdf]

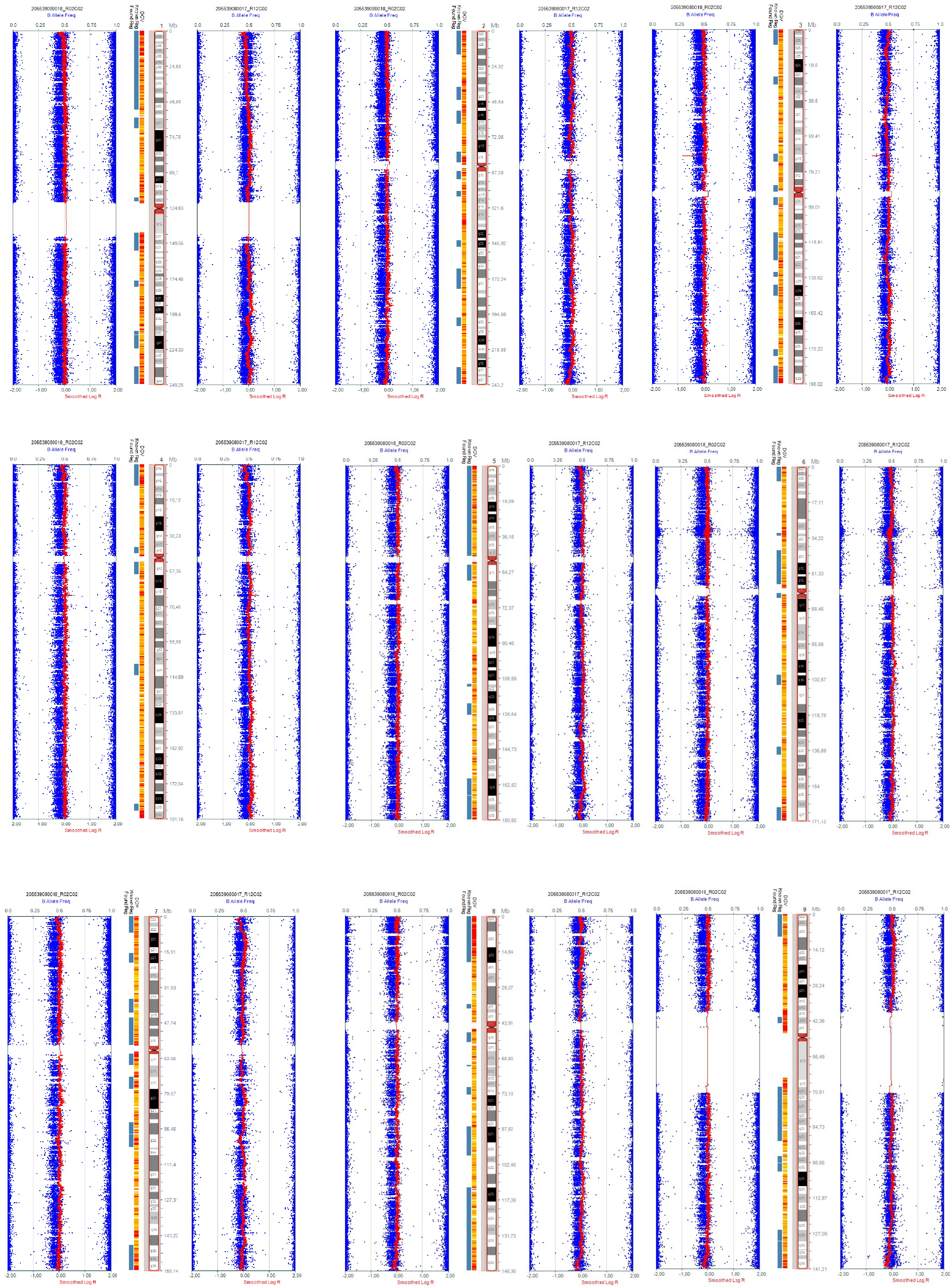

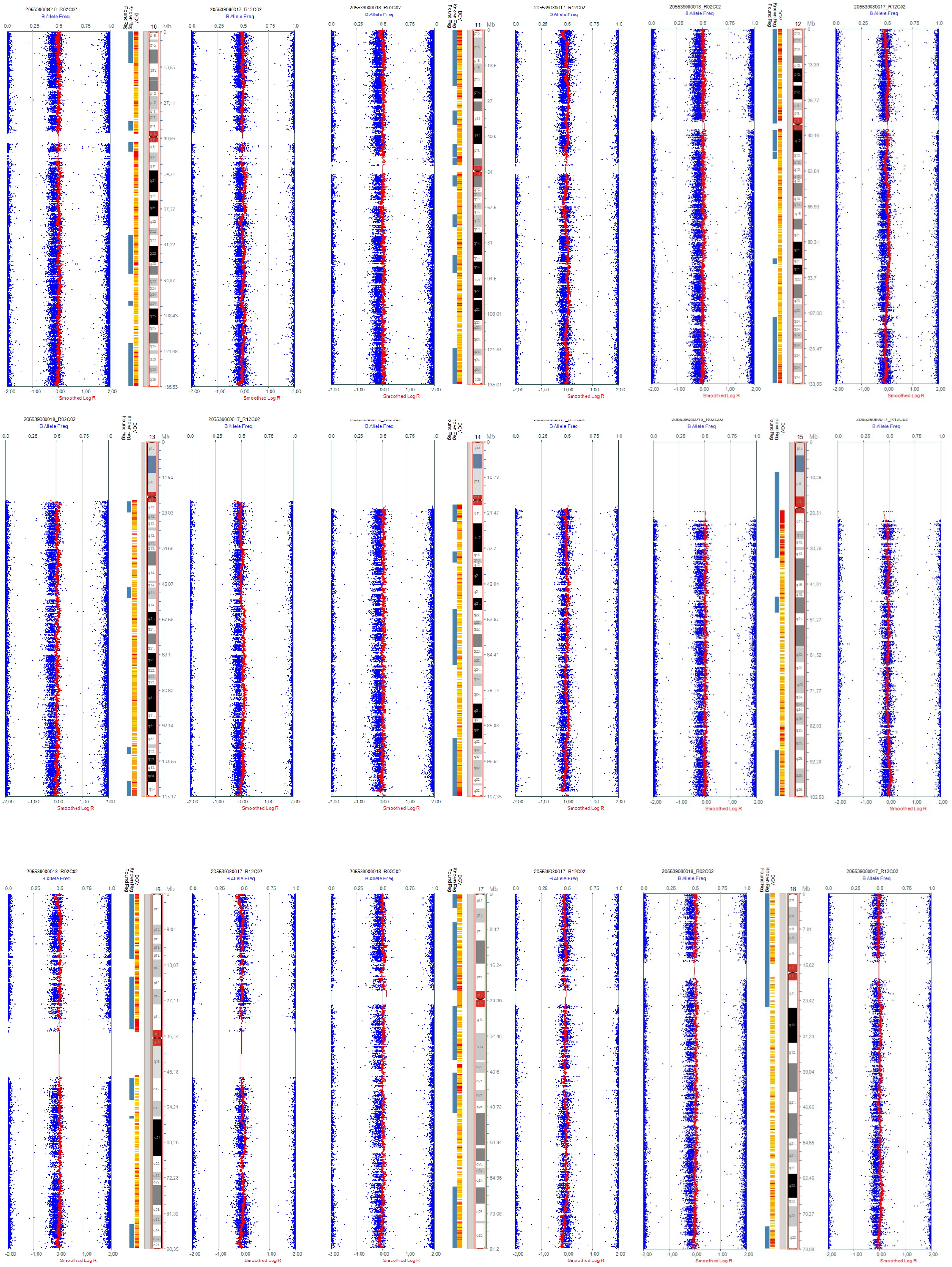
205539080018_R02C02
205539080017_R12C02
B Allele Freq
Smoothed Log R
DGV
Known Reg
Found Reg
Mb
10
11
12
13
14
15
16
17
18

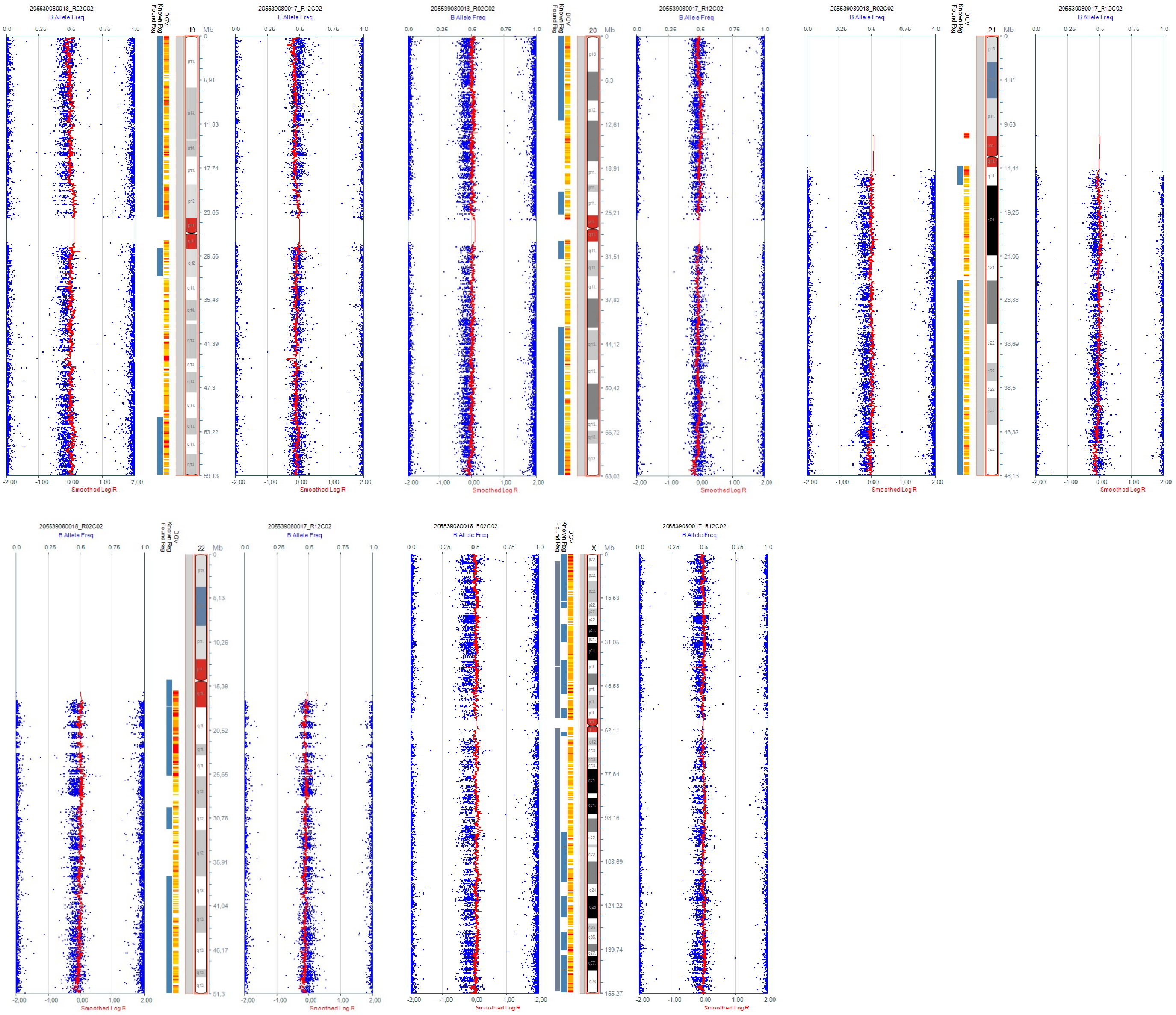

Supplementary Fig. S1: B-allele frequency and Smoothed LogR ration plot for individual chromosomes for WA25 (R12C02) and C3 (R02C02) hESC lines as analyzed in KaryoStudio v.1.4.
