# Supplementary material for: Generation of a Well-Characterized Homozygous Chromodomain-Helicase-DNA-Binding Protein 4G1003D Mutant hESC Line Using CRISPR/eCas9 (ULIEGEe001-A-1)
Source: Int J Mol Sci. 2023 Jun 23;24(13):10543. doi: 10.3390/ijms241310543 (PMC10342000; doi:10.3390/ijms241310543)
Supplement: Supplementary file 1 [file ijms-24-10543-s001.zip › ijms-2444738-Figure S2-S4.pdf]

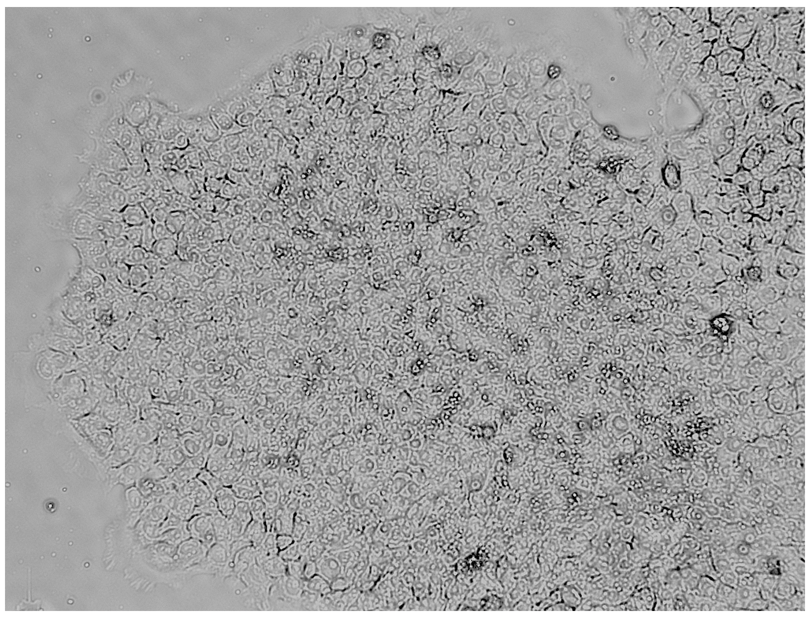

Supplementary Fig. S2: Bright field image of C3 cell line.

Luminescence Reading	Luminescence Value	Ratio (B/A) Result
A	789	0.292
B	231	

Supplementary Fig. S3: Mycoplasma testing results.

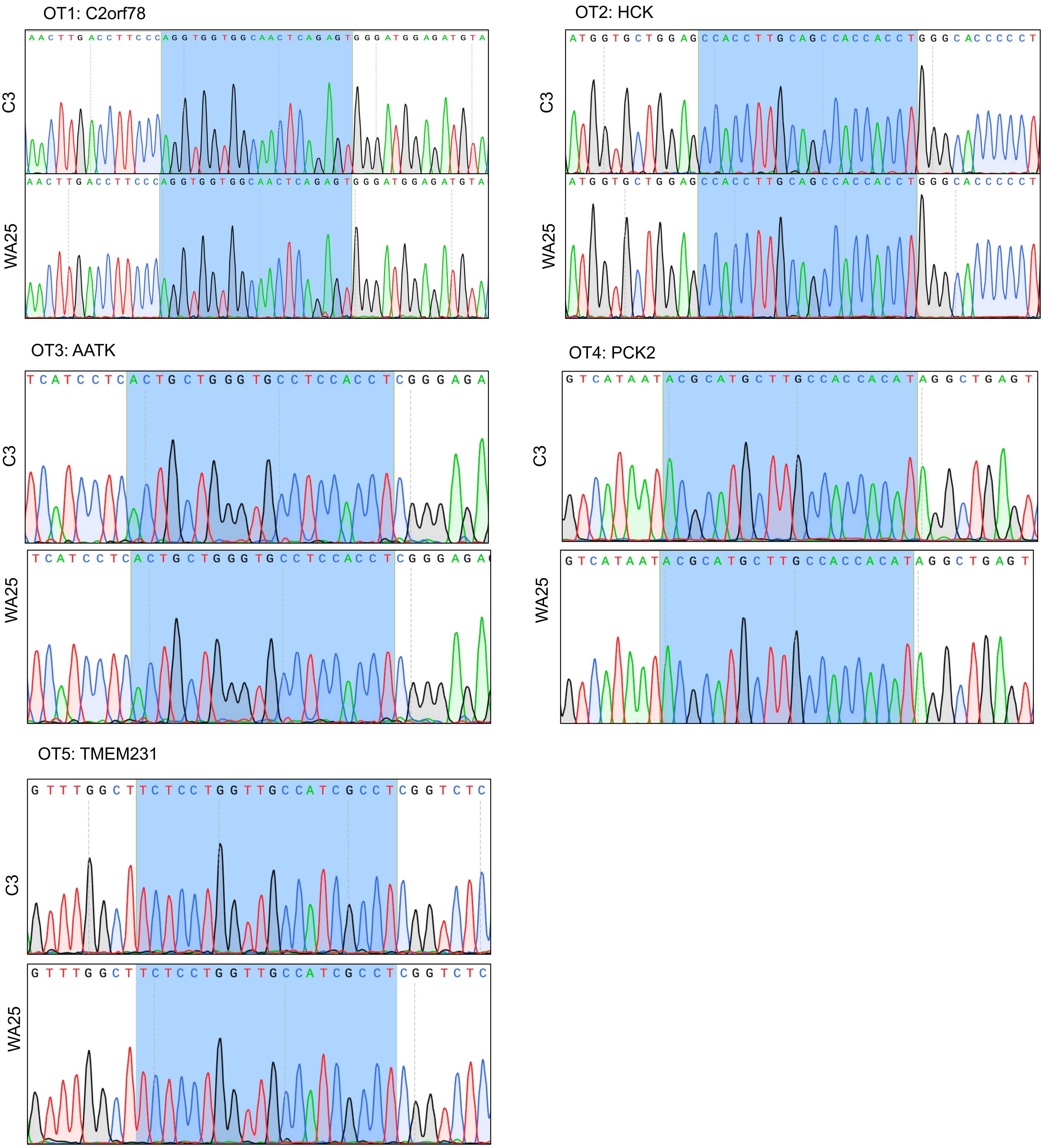

Supplementary Fig. S4: Sanger sequencing chromatogram of top five exonic off-targets from wild-type WA25 and edited C3 hESC cell lines.
